# Developing physical Activity and Sedentary behaviour thresholds for the Secondary prevention of Heart disease (DASSH): a cohort mortality survival tree analysis

**DOI:** 10.1186/s12966-025-01743-6

**Published:** 2025-04-10

**Authors:** Nicole Freene, Amanda Lönn, Theo Niyonsenga, Suzanne Carroll, Adrian Bauman, Robyn Gallagher, Rachel Davey

**Affiliations:** 1 Health Research Institute, University of Canberra, Bruce, ACT 2617 Australia; 2 Department of Physical Activity and Health, The Swedish School of Sport and Health Sciences, Gymnastik- Och Idrottshögskolan (GIH), Lidingövägen 1, Stockholm, 114 33 Sweden; 3 Sydney School of Public Health, Faculty of Medicine and Health, The University of Sydney, Sydney, NSW 2600 Australia; 4 Charles Perkins Centre, Susan Wakil School of Nursing and Midwifery, Sydney Nursing School, Faculty of Medicine and Health, The University of Sydney, Sydney, NSW 2600 Australia

**Keywords:** Cardiology, Lifestyle behaviours, Public health

## Abstract

**Background:**

The dose–response relationship between physical activity and sedentary behaviour (SB) with mortality in people with coronary heart disease (CHD) is unclear. The aim was to identify moderate-to-vigorous physical activity (MVPA) and SB thresholds for mortality risk.

**Methods:**

This prospective cohort study comprised Australian participants aged ≥ 45 years with self-reported CHD (2006–2020). Self-reported MVPA (min/wk) and SB (hr/day) were the exposures. Cardiac and all-cause mortality were the main outcomes. Survival regression trees identified MVPA and SB thresholds influencing mortality survival rate. Cox regression models and the C-statistic were used to examine the thresholds, comparing them to public health guidelines.

**Results:**

The cohort included 40,156 participants (mean (SD) age, 70.3(10.3) years; 15,278 females (38%)). During a median follow-up of 11.1 (IQR,6.2–14.4) years, 2,497 cardiac and 12,240 all-cause deaths were recorded. The threshold for MVPA and all-cause and cardiac mortality was ≥ 146 min/wk and ≥ 96 min/wk, respectively. For SB, the threshold for mortality was < 5–6 h/day. Sex-specific differences in thresholds for MVPA and SB were found. All MVPA and SB thresholds had equivalent associated risk reductions and predictive abilities for cardiac and all-cause mortality to the public health guidelines.

**Conclusion:**

The newly identified thresholds suggest that the public health physical activity guidelines are suitable for reducing risks of all-cause mortality in people with CHD. For reducing risks of cardiac mortality, the threshold is suggested to be much lower. The SB suggested thresholds for reducing risks of mortality are 5–6 h/day. Further research is required to explore these thresholds and sex-specific differences.

**Supplementary Information:**

The online version contains supplementary material available at 10.1186/s12966-025-01743-6.

## Background

Physical inactivity is an independent risk factor for cardiovascular disease (CVD) and all-cause mortality, in healthy and CVD populations [1, 2]. The public health physical activity guidelines recommend that adults with and without chronic disease should achieve at least 150-min of moderate-intensity aerobic physical activity, or at least 75-min of vigorous-intensity aerobic physical activity, or an equivalent combination of both throughout the week, and avoid long periods of sedentary time to improve health outcomes [3]. For people who have coronary heart disease (CHD), international clinical guidelines recommend that individuals should aim to achieve the public health physical activity guidelines for secondary prevention of CVD [4–6]. Studies assessing physical activity in people with CHD have established an inverse relationship between increased levels of physical activity and mortality using self-reported physical activity data [7–10], although, the exact dose–response relationship between physical activity and sedentary behaviour with cardiac and all-cause mortality is less definite.

Individuals with CHD are at a much higher risk of death compared to healthy individuals [11]. Despite this, physical activity levels remain low and sedentary behaviour is high in this population [12, 13]. The current public health physical activity guidelines may represent a barrier for people with CHD who do not attempt to reach this threshold as it is perceived too difficult to achieve [14]. Reducing sedentary behaviour may be a more achievable first-line strategy for cardiac patients, moving participants along the energy expenditure continuum, aiming to increase their physical activity levels over the medium-to-longer term. However, it is unclear how long people with CHD should limit their sedentary time to, or how much physical activity they should be doing to reduce the risk of cardiac and all-cause mortality.

Currently, there are no disease-specific physical activity and sedentary behaviour guidelines for people with CHD and people with CHD have not been considered in international physical activity guidelines [15]. The development of disease-specific physical activity and sedentary behaviour guidelines will guide clinical practice and research, improving health outcomes for this population. Therefore, the aims of this study are to: (i) identify moderate-to-vigorous physical activity (MVPA) and sedentary behaviour thresholds influencing cardiac and all-cause mortality survival rate; (ii) examine the dose–response relationship between the newly identified MVPA and sedentary behaviour thresholds with cardiac and all-cause mortality; and (iii) compare the newly identified and public health MVPA and sedentary behaviour thresholds in terms of their predictive ability in a cohort of middle-aged and older Australian adults with self-reported CHD.

## Methods

### Study population

The 45 and Up Study is a large-scale prospective study of 267,151 Australian men and women aged ≥ 45 years, randomly sampled from the general population of New South Wales (NSW), Australia [16]. Individuals joined the study by completing a postal questionnaire and giving informed consent for follow-up through repeated data collection and linkage of their data to population health databases. The current analysis was based on participants who identified themselves as having ‘heart disease as diagnosed by a doctor’, or being treated for a ‘heart attack or angina’ or ‘other heart disease’ in the last month, or had a ‘coronary artery bypass graft operation’ in the first (2006 to 2009), Social, Economic and Environmental Factors (SEEF) (2010), second (2012–2016) or third (2018–2020) survey wave. The participants ‘baseline’ data was defined as the wave where they first reported CHD. The dose–response association of physical activity and sedentary behaviour with mortality risk for this cohort has been reported elsewhere [17].

### Exposures

#### Physical activity

MVPA in the last week was measured across all waves using the Active Australia Survey (AAS) [18]. For each activity type (walking continuously for at least 10 min (walking), vigorous physical activity (VPA), other moderate physical activity (MPA)) participants were asked two questions (6 questions in total): the number of sessions, and the time completed in minutes or hours in the last week, using a continuous scale (Supplementary Table 1). The AAS has been reported as reliable and of acceptable validity in people with CHD [19]. Total time in MVPA was calculated using formula: walking + MPA + (2 × VPA) [18]. The total time (min/wk) in MVPA was limited to 1680 min, or 840 min for a single activity type [18]. If participants responded to at least one AAS question, 0 min were assigned for missing activity type.

#### Sedentary behavior

Sedentary behaviour questions varied across the waves (Supplementary Table 1). In the first and SEEF waves, participants were asked how many hours in each 24-h day they spent: ‘sitting’ or ‘watching TV/using a computer’ and these questions were not mutually exclusive, that is, ‘sitting’ did not exclude the hours spent ‘watching TV/using a computer’. In waves 2 and 3 participants were asked ‘how much time they spent in the last 7 days on a usual weekday and weekend day sitting for: transport; work; watching TV; using computer at home; other leisure activities’. The average daily time in total sedentary behavior in waves 2 and 3 were calculated using formula: (weekday total sedentary behaviour × 5 + weekend total sedentary behaviour × 2)/7. If participants responded to at least one sedentary behavior question, 0 min were recorded for missing sedentary behavior domain minutes. Total sedentary time was limited to 16 h per day.

### Outcomes

The outcome variables, cardiac mortality and all-cause mortality, were obtained from the NSW Register of Births, Deaths & Marriages – Death Registrations and the Australian Bureau of Statistics Mortality Data (Cause of Death Unit Record File) [20] from the participant’s ‘baseline’ survey to 31 December 2022. Cardiac mortality was defined according to the International Statistical Classification of Disease and Related Health Problems 10th Revision (ICD-10) codes I20-I25 and I46.2 [21].

### Covariates

The covariates included were based on CVD risk factors and physical activity and sedentary behaviour correlates [22], including age, sex (male, female), education level (less than high school, high school, tertiary education), body mass index (BMI; kg/m^2^), smoking status (current, not current), type 2 diabetes as diagnosed by a doctor (yes, no) and family history of heart disease (yes, no; for mother/father/brother/sister) self-reported in the participants ‘baseline’ questionnaire [16].

### Data analysis

To be included in the analyses, individuals needed to have complete data for exposures, outcomes and covariates. Descriptive demographic characteristics were presented as frequencies, medians and interquartile ranges, and means and standard deviations. Baseline differences in demographic characteristics between males vs females, survivors vs non-survivors and included vs excluded individuals were analyzed using χ2, Mann- Whitney U, and paired t-tests. The follow-up time was calculated from the date of answering the ‘baseline’ questionnaire to the date of death or the end of the follow-up period (31 December 2022), whichever came first. *P*-values < 0.05 were considered statistically significant.

The Survival Regression Tree approach was used to develop thresholds for MVPA (min/wk) and sedentary behaviour (hr/day) relating it to risk of cardiac and all-cause mortality. Cardiac and all-cause mortality were coded as binary variables (i.e.: ‘yes/no’) and survival regression tree models were fitted to the data for physical activity and sedentary behaviour separately (min split = 20, complexity parameter = 0.001, max depth = 1). The goal of the survival regression tree was to identify homogenous sub-groups, characterised by prognostic variables (i.e. physical activity and sedentary behaviour baseline variables) in a heterogeneous population on the basis of the risk or probability of cardiac and all-cause mortality, enabling classification by prognosis [23]. The survival tree algorithm selects the variable value (i.e. MVPA min/wk or sedentary behaviour hr/day) with the highest ability to separate survivors and non-survivors using *p*-values from permutation distributions. The homogeneity in the survival regression tree refers to the absence of sufficient statistical evidence of variation in time-to-event distribution [23, 24]. Survival regression trees were completed for the total cohort, and males and females separately to explore differences in sex.

To examine the newly identified thresholds for MVPA and sedentary behavior with mortality, several Cox regression models were built. Hazard ratios (HRs) and their 95% confidence interval were computed, and the HRs were considered statistically significant if the 95% confidence interval did not include the value of 1. The HRs for MVPA and sedentary behaviour (binary categorical data) were analyzed using unadjusted and fully adjusted (all covariates included) models. All MVPA analyses were adjusted for sedentary behaviour and the sedentary behaviour analyses were adjusted for MVPA. Cox regression models were completed for the total sample, and males and females separately to explore differences based on sex. Sensitivity analyses were performed for all models to reduce the risk of including individuals with a long history of pre-existing CHD by excluding wave 1 individuals. Additionally, HRs [95% CI] for MVPA and sedentary behavior binary categories based on the public health physical activity guidelines [3] (≥ 150 min/wk MVPA, < 150 min/wk MVPA) and a suggested general public sedentary behaviour threshold [25] (≥ 7 h/day sedentary behaviour, < 7 h/day sedentary behaviour) were analyzed for the total sample to assess whether associations were similar to the newly identified thresholds for MVPA and sedentary behaviour. The C statistic, which describes the area under the receiver operating characteristic (ROC) curve, was used to evaluate and compare the newly identified and public health/general public thresholds in terms of their predictive ability. A score above 0.50 shows that the model has some predictive power. In general, the thresholds with the higher area under the ROC curve may be considered the better thresholds [26]. Sensitivity and specificity analyses were used to identify the proportion of true positive and true negative mortality events. All statistical analyses were performed using R version 2023.03.1 (R Core Team 2023), using the packages rpart, rpart.plot, survival and proc.

## Results

Between 2006 and 2020 49,828 participants in the 45 and Up Study self-reported CHD. After excluding participants who did not have complete data for exposures, outcomes and covariates, 40,156 participants were included in the analysis (Supplementary Fig. 1 and Supplementary Table 2). At baseline the average age of participants was 70 ± 10 years (Table 1). The majority were male (62%), overweight, with no tertiary education (78%). Six in ten had a family history of heart disease. Only 4% were current smokers and 16% had type 2 diabetes. The median time spent in sedentary behaviour was 5 h/day, with women reporting less sitting. High levels of MVPA were self-reported (6.5 h/wk), with men reporting more MVPA than women. The median follow-up time was 11 years, with 30% of participants dying of any cause and 6% dying due to a cardiac cause. It was less common for females, participants who were younger, tertiary educated, had type 2 diabetes and did not have a family history of heart disease to die during follow-up (Supplementary Table 3).

**Table 1:** Descriptive characteristics of participants at their baseline (first report of coronary heart disease)

Characteristic	Males (*n* = 24,878)	Females (*n* = 15,278)	Total (*n* = 40,156)
Age *(yr)*, mean (SD)	70.4 (9.9)	70.0 (10.8)	70.25 (10.25)
Tertiary education, number (%)	5901 (23.7)	2781 (18.2)	8682 (21.6)
Type 2 diabetes, number yes (%)	4348 (17.5)	2187 (14.3)	6535 (16.3)
Body mass index *(kg/m*^*2*^*)*, mean (SD)	27.26 (4.18)	27.20 (5.47)	27.24 (4.71)
Family history heart disease, number yes (%)	14,801 (59.5)	10,269 (67.2)	25,070 (62.4)
Current smokers, number yes (%)	1119 (4.5)	665 (4.4)	1784 (4.4)
Sedentary behavior total (*hr/day*), median (IQR)	5 (3–7)	3 (4–6)	5 (3–7)
MVPA (*min/wk*), median (IQR)	390 (150–840)	380 (120–840)	390 (140–840)
Walking (*min/wk*), median (IQR)	120 (30–240)	90 (20–210)	100 (30–240)
Moderate physical activity (*min/wk*), median (IQR)	120 (10–360)	150 (10–420)	120 (10–403)
Vigorous physical activity (*min/wk*), median (IQR)	0 (0–60)	0 (0–20)	0 (0–40)
Follow-up time (*days*), median (IQR)	4179 (2241–5255)	3899 (2294–5251)	4084 (2261–5255)
All-cause mortality, number deaths (%)	8404 (33.8)	3836 (25.1)	12,240 (30.5)
Cardiac mortality, number deaths (%)	1790 (7.2)	707 (4.6)	2497 (6.2)

*MVPA* Moderate-to-Vigorous Physical Activity

**Fig. 1:**
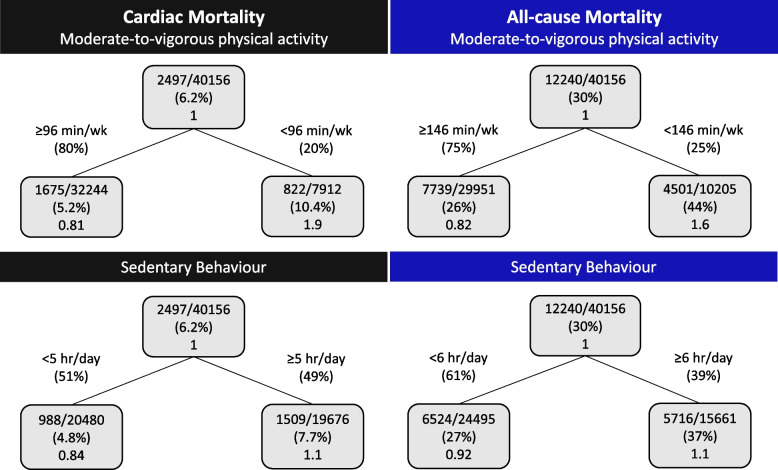
Survival regression trees for cardiac and all-cause mortality as a function of moderate-to-vigorous physical activity and sedentary behaviour in adults with coronary heart disease (*n* = 40,156). Inside each box the top line is the number of events divided by the number of individuals, the second line is the percentage and the third line is the hazard ratio compared to the parent node

**Fig. 2:**
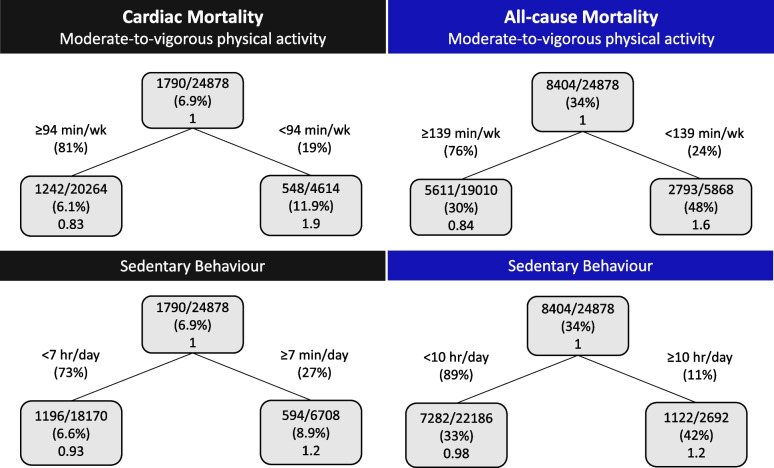
Survival regression trees for cardiac and all-cause mortality as a function of moderate-to-vigorous physical activity and sedentary behaviour in males with coronary heart disease (*n* = 24,878). Inside each box the top line is the number of events divided by the number of individuals, the second line is the percentage and the third line is the hazard ratio compared to the parent node

**Fig. 3:**
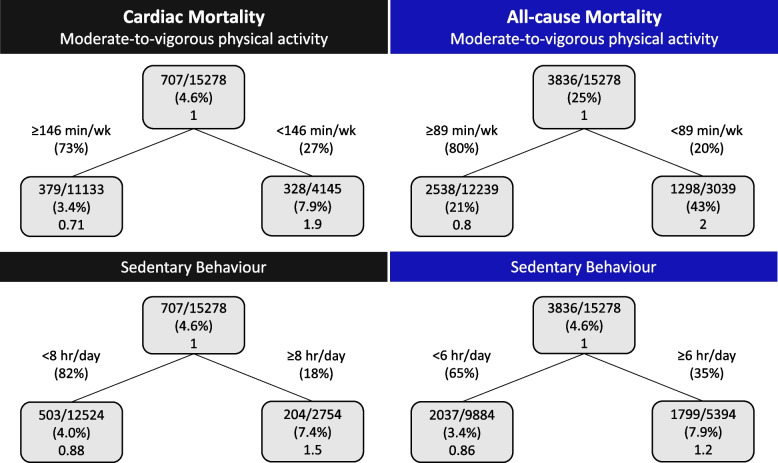
Survival regression trees for cardiac and all-cause mortality as a function of moderate-to-vigorous physical activity and sedentary behaviour in females with coronary heart disease (*n* = 15,278). Inside each box the top line is the number of events divided by the number of individuals, the second line is the percentage and the third line is the hazard ratio compared to the parent node

The survival regression tree thresholds for cardiac and all-cause mortality as a function of MVPA and sedentary behaviour in the total cohort, males, and females are shown in Figs. 1, 2, and 3. Figure 1 shows individuals that reported ≥ 96 min/wk MVPA had 94.8% survival probability for cardiac mortality, while individuals that reported < 96 min/wk MVPA had 89.6% survival probability. For all-cause mortality, if individuals reported ≥ 146 min/wk MVPA they had a 74% survival probability, while individuals that reported < 146 min/wk MVPA had a 56% survival probability. Sedentary behaviour < 5 h/day was found to have a survival probability for cardiac mortality of 95.2%, while ≥ 5 h/day has a survival probability of 92.3%. For all-cause mortality, if individuals reported < 6 h/day sedentary behaviour they had a 73% survival probability, while individuals that reported ≥ 6 h/day sedentary behaviour had a 64% survival probability. The survival probabilities for males and females were similar to the total cohort, yet the newly identified thresholds for MVPA and sedentary behaviour varied between the total cohort, males and females (Figs. 1, 2, and 3).

Using the newly identified thresholds, adjusted Cox regression analyses found a 35% lower risk of all-cause mortality was associated with 146 min/wk or more of MVPA compared to less than 146 min/wk (Table 2). The new MVPA threshold was similar for males with ≥ 139 min/wk MVPA reducing the associated risk of dying from all-causes by 34% (Table 3). In contrast, in females the threshold was lower, ≥ 89 min/wk, but the associated risk reduction was similar at 37% for all-cause mortality (Table 4). For cardiac mortality, 96 min/wk or more of MVPA decreased associated risk by 40% compared to less than 96 min/wk (Table 2). This was similar for males but for females, the threshold for MVPA was higher, ≥ 146 min/wk, to achieve a similar associated risk reduction in cardiac mortality (Tables 3 and 4). Sensitivity analyses confirmed the main analyses for MVPA and removing Wave 1 did not change the direction of the results, although, the threshold for cardiac mortality was lower resulting in a higher associated risk reduction (Supplementary Table 4).

**Table 2:** Hazard ratios [95% CI] for cardiac and all-cause mortality by moderate-to-vigorous physical activity and sedentary behaviour in adults with coronary heart disease (*n* = 40,156)

	n	n cases	Unadjusted model	Adjusted model^a^	Area under ROC curve^d^	Sensitivity^d^	Specificity^d^
Moderate-to-vigorous physical activity^b^							
All-cause mortality, < 146 min/wk	10,205	4501	Ref	Ref	0.582	0.368	0.204
All-cause mortality, ≥ 146 min/wk	29,951	7739	0.503 [0.485; 0.522]	0.653 [0.629; 0.679]			
Cardiac mortality, < 96 min/wk	7912	822	Ref	Ref	0.532	0.310	0.246
Cardiac mortality, ≥ 96 min/wk	32,244	1675	0.420 [0.386–0.456]	0.606 [0.556; 0.661]			
Sedentary behaviour^c^							
All-cause mortality, ≥ 6 h/day	15,661	5716	Ref	Ref	0.570	0.329	0.188
All-cause mortality, < 6 h/day	24,495	6524	0.829 [0.797; 0.861]	0.844 [0.812;;0.877]			
Cardiac mortality, ≥ 5 h/day	19,676	1509	Ref	Ref	0.544	0.485	0.398
Cardiac mortality, < 5 h/day	20,480	988	0.778 [0.719; 0.842]	0.812 [0.751; 0.880]			

*ROC* Receiver Operating Characteristic

^a^All models adjusted for age, sex, education level, body mass index, smoking, type 2 diabetes, family history of heart disease

^b^Model also adjusted for Sedentary Behaviour

^c^Model also adjusted for Moderate-to-Vigorous Physical Activity

^d^Based on the unadjusted model

**Table 3:** Hazard ratios [95% CI] for cardiac and all-cause mortality by moderate-to-vigorous physical activity and sedentary behaviour in males with coronary heart disease (*n* = 24,878)

	n	n cases	Unadjusted model	Adjusted model^a^	Area under ROC curve^d^	Sensitivity^d^	Specificity^d^
Moderate-to-vigorous physical activity^b^							
All-cause mortality, < 139 min/wk	5868	2793	Ref	Ref	0.573	0.332	0.187
All-cause mortality, ≥ 139 min/wk	19,010	5611	0.529 [0.505; 0.553]	0.662 [0.631; 0.693]			
Cardiac mortality, < 94 min/wk	4614	548	Ref	Ref	0.565	0.306	0.176
Cardiac mortality, ≥ 94 min/wk	20,264	1242	0.431 [0.390; 0.477]	0.596 [0.537; 0.661]			
Sedentary behaviour^c^							
All-cause mortality, ≥ 10 h/day	2692	1122	Ref	Ref	0.508	0.073	0.057
All-cause mortality, < 10 h/day	22,186	7282	0.809 [0.745; 0.879]	0.752 [0.693; 0.818]			
Cardiac mortality, ≥ 7 h/day	6708	594	Ref	Ref	0.526	0.277	0.225
Cardiac mortality, < 7 h/day	18,170	1196	0.806 [0.727; 0.894]	0.772 [0.695; 0.857]			

*ROC* Receiver Operating Characteristic

^a^All models adjusted for age, sex, education level, body mass index, smoking, type 2 diabetes, family history of heart disease

^b^Model also adjusted for Sedentary Behaviour

^c^Model also adjusted for Moderate-to-Vigorous Physical Activity

^d^Based on the unadjusted model

**Table 4:** Hazard ratios [95% CI] for cardiac and all-cause mortality by moderate-to-vigorous physical activity and sedentary behaviour in females with coronary heart disease (*n* = 15,278)

	n	n cases	Unadjusted model	Adjusted model^a^	Area under ROC curve^d^	Sensitivity^d^	Specificity^d^
Moderate-to-vigorous physical activity^b^							
All-cause mortality, < 89 min/wk	3039	1298	Ref	Ref			
All-cause mortality, ≥ 89 min/wk	12,239	2538	0.403 [0.377; 0.431]	0.629 [0.586; 0.675]	0.593	0.338	0.152
Cardiac mortality, < 146 min/wk	4145	328	Ref	Ref			
Cardiac mortality, ≥ 146 min/wk	11,133	379	0.374 [0.323; 0.434]	0.617 [0.528; 0.721]	0.601	0.464	0.262
Sedentary behaviour^c^							
All-cause mortality, ≥ 6 h/day	5394	1799	Ref	Ref			
All-cause mortality, < 6 h/day	9884	2037	0.715 [0.668; 0.766]	0.803 [0.749; 0.861]	0.546	0.311	0.218
Cardiac mortality, ≥ 8 h/day	2754	204	Ref	Ref			
Cardiac mortality, < 8 h/day	12,524	503	0.557 [0.461; 0.673]	0.746 [0.614; 0.907]	0.537	0.188	0.115

*ROC* Receiver Operating Characteristic

^a^All models adjusted for age, sex, education level, body mass index, smoking, type 2 diabetes, family history of heart disease

^b^Model also adjusted for Sedentary Behaviour

^c^Model also adjusted for Moderate-to-Vigorous Physical Activity

^d^Based on the unadjusted model

For sedentary behaviour, adjusted Cox regression analyses found less than 6 h/day of sedentary behaviour was associated with a 16% lower risk of all-cause mortality compared to 6 h/day or more of sedentary behaviour (Table 2). For males, the threshold for sedentary behaviour was higher, < 10 h/day, and was associated with a higher risk reduction in all-cause mortality at 25% (Table 3). Females had the same threshold for sedentary behaviour as the total sample, with a slightly greater risk reduction for all-cause mortality, 20% (Table 4). For cardiac mortality, less than 5 h/day of sedentary behaviour was associated with a 19% lower risk compared to 5 h or more of sedentary behaviour (Table 2). Males and females had higher thresholds for sedentary behaviour, less than 7 and 8 h/day respectively, with associated risk reductions in cardiac mortality of 23–25% (Tables 3 and 4). Sensitivity analyses for sedentary behaviour provided substantially lower thresholds and HRs with wide confidence intervals, indicating large variability in sedentary behaviour when removing Wave 1 and providing little knowledge about the effect (Supplementary Table 4).

The public health physical activity guidelines binary categories yielded similar results for MVPA associated risk reduction for cardiac and all-cause mortality, even though the newly identified MVPA threshold to reduce associated cardiac mortality risk was substantially lower than the public health guidelines, 96 vs 150 min/wk MVPA (Table 2, Supplementary Table 5). The newly identified threshold for sedentary behaviour was also lower compared to the general public sedentary behaviour categories, 5–6 vs 7 h/day (Table 2, Supplementary Table 5). Using the newly identified thresholds, all models had some predictive power with the area under the ROC curve’s > 0.50 but low sensitivity and specificity (Tables 2, 3, and 4). Comparing the predictive power of the newly identified thresholds to the public health thresholds (Supplementary Table 5), the results were similar suggesting that the new thresholds were no better or worse than the currently used public health guidelines in this cardiac population.

## Discussion

In people with CHD the newly identified MVPA threshold for cardiac mortality was 34% lower than the public health physical activity guidelines. However, the newly identified threshold for MVPA and all-cause mortality was the same as the public health physical activity guidelines. The newly identified sedentary behaviour threshold was also 1–2 h per day lower than the general public sedentary behaviour threshold for both cardiac and all-cause mortality. Interestingly, there were sex differences in identified thresholds for MVPA and sedentary behaviour for cardiac and all-cause mortality. For all-cause mortality, MVPA appeared more protective for females and sedentary behaviour more protective for men, that is, females needed to limit their sedentary behaviour more than males to receive similar all-cause mortality risk reductions. The opposite was true for cardiac mortality. Importantly, all newly identified MVPA and sedentary behaviour thresholds had equivalent associated risk reductions and predictive abilities for cardiac and all-cause mortality to the current public health physical activity guidelines and general public sedentary behaviour thresholds but clinically, the new thresholds may be easier to achieve for people with CHD.

Self-reported physical activity was high and sedentary behaviour was low for people with CHD as found elsewhere [8, 12], with the mortality rate similar to other cohorts [27]. Similar associated risk reductions were found for all-cause mortality when people with CHD met the public health physical activity guidelines (≥ 150 min/wk MVPA) ranging from 19 – 44% [8, 9, 28, 29], comparable to general population cohorts [30], and similar to our newly identified threshold of ≥ 146 min/wk MVPA. To our knowledge, only one other study has investigated the dose–response relationship of MVPA with cardiac mortality in people with CHD, reporting that when individuals met the public health physical activity guidelines, their reduced associated risk of cardiac mortality was the same as found here (37%) [7]. This study also found that MVPA was more markedly inversely associated with cardiac mortality than all-cause mortality, supporting a lower threshold of MVPA needed to achieve the same associated risk reduction as found in our study. In contrast, in the general population, the MVPA associations with both CVD and all-cause mortality are similar [15]. This is clinically relevant for the secondary prevention of CHD as the new threshold of ≥ 96 min/wk MVPA should be easier to achieve for people with CHD compared to the public health physical activity guidelines, potentially reducing the risk of disease-specific mortality.

In the general population, the suggested threshold for sedentary behaviour and all-cause mortality is 7 h/day [25], although, the evidence is currently deemed insufficient to set time-based public health recommendations for sedentary time [31]. The newly identified sedentary behaviour threshold for people with CHD is 1 h/day lower for all-cause mortality and 2 h/day lower for cardiac mortality. This indicates that limiting sedentary behaviour may be more important in people with CHD compared to the general population, and particularly important for disease-specific mortality. This is different to physical activity, where less MVPA resulted in similar associated risk reductions in cardiac mortality compared with meeting the public health physical activity guidelines. No other studies have investigated the dose–response relationship between sedentary behaviour with both cardiac and all-cause mortality in people with CHD. Sedentary behaviour guidance is important in people with CHD especially if they find it difficult to meet the physical activity guidelines. Reducing sedentary behaviour may be an achievable first line strategy in their recovery from a cardiac event, noting that sedentary individuals have the most to gain with low volumes of MVPA resulting in substantial health benefits [17].

There is evidence from our study that the relationship between MVPA and sedentary behaviour with cardiac and all-cause mortality varies by sex in people with CHD. In females compared with males, MVPA appears to be more protective for all-cause mortality, that is females don’t need to complete as much MVPA as males to achieve a similar associated risk reduction. However, MVPA for females appears to be less protective for cardiac mortality. In males compared to females, reduced sedentary behaviour appears to be more protective, that is males don’t need to limit their sedentary behaviour as much as females to achieve a similar associated risk reduction for all-cause mortality but slightly less protective for cardiac mortality. In the general population, the dose–response relationship between MVPA with all-cause and cardiovascular disease mortality has been reported as not varying by sex [15]. Conversely, a recent large prospective cohort study found that females compared with males had greater gains in all-cause and cardiovascular mortality risk reduction from equivalent doses of physical activity [32]. This has also been reported in a CHD prospective cohort study [10] and is similar to our finding for all-cause mortality but not cardiac mortality. Together, these results highlight a sex differential response in both a CHD and general population. Less physical activity is potentially needed for females to get the same health benefits, which may be useful to engage females in physical activity as they commonly report less MVPA than males [33]. Factors that may contribute to the sex-specific differences in the physical activity thresholds may be the severity of CHD as it is known that CHD in women is currently under-recognised, under-diagnosed, under-treated and under-researched resulting in women less likely to present and receive appropriate treatment during and after a myocardial infarction [34, 35]. Additionally, it is well known that men have a greater exercise capacity than women due to physiological differences which may affect this realtionship [36]. Thus, further investigation of sex-specific differences and cardiac-specific mortality in this population is indicated to determine the dose–response relationship of physical activity and sedentary behaviour, allowing improved guidance for the secondary prevention of CHD.

### Strengths and limitations

This is the first study to investigate new MVPA and sedentary behaviour thresholds and their relationship with mortality in a large prospective cohort of people with CHD using survival regression trees. Additional strengths are the use of registry data to determine mortality outcomes, the use of the public health physical activity guidelines for comparison, investigation of sex-specific differences and sensitivity analyses. Nevertheless, our study is not without limitations. Self-reported CHD has not been validated in this cohort. Although internationally, including Australia, self-reported history of CHD has been found to be a valid measure of diagnosed CHD in population-based studies [37–40]. Both physical activity and sedentary behaviour were self-reported and subject to recall and social desirability bias [19, 41–43], and the sedentary behaviour questions changed between waves. Women and men may also self-report physical activity differently using the AAS with some evidence that men over-report MVPA resulting in lower associations with device measures [44], although another study found sex was not associated with self-report bias [45]. Most non-modifiable and some modifiable CVD risk factors were included as covariates but not all, including hypertension, and those not included may be important factors in disease-specific and all-cause mortality. Participants excluded from our cohort were older, less likely to be female and tertiary educated, more likely to die from cardiac or any cause, and had higher levels of type 2 diabetes and lower levels of MVPA so results may not be generalizable to this population, or other countries as this study was only conducted in Australia with individuals that were racially and ethnically homogenous. Finally, the 45 and Up study tends to include healthier participants compared to the general population, although another study of the same population found similar estimates of exposure-outcome relationships [46].

## Conclusion

The public health physical activity guidelines to decrease the probability of all-cause mortality appear to be applicable to people with CHD. However, the threshold of MVPA may be substantially lower to decrease the risk of cardiac mortality, and therefore, easier to achieve for people with CHD. Additionally, the threshold for sedentary behaviour appears to be 5–6 h/day for cardiac and all-cause mortality, which may be more difficult to achieve than the current general public 7 h/day recommendation. Sex-specific differences in MVPA and sedentary behaviour should be considered and further investigation is required to explore the newly identified thresholds to reduce the risk of cardiac and all-cause mortality in people with CHD.

## Supplementary Information

Supplementary Material 1.

Supplementary Material 2.

## Data Availability

The datasets used and analysed during the study are not available as they are third party data not owned or collected by the authors. The data are available from the data custodians for approved research projects. Data access enquiries can be made to the Sax Institute https://www.saxinstitute.org.au/solutions/45-and-up-study/use-the-45-and-up-study/apply-for-access/

## Abbreviations

AAS: Active Australia Survey
BMI: Body mass index
CHD: Coronary heart disease
CVD: Cardiovascular disease
HR: Hazard ratio
MPA: Moderate physical activity
MVPA: Moderate-to-vigorous physical activity
NSW: New South Wales
SEEF: Social, economic and environmental factors
VPA: Vigorous physical activity
