# Supplementary figures and images for: Developing physical Activity and Sedentary behaviour thresholds for the Secondary prevention of Heart disease (DASSH): a cohort mortality survival tree analysis
Source: Int J Behav Nutr Phys Act. 2025 Apr 10;22:43. doi: 10.1186/s12966-025-01743-6 (PMC11987228; doi:10.1186/s12966-025-01743-6)

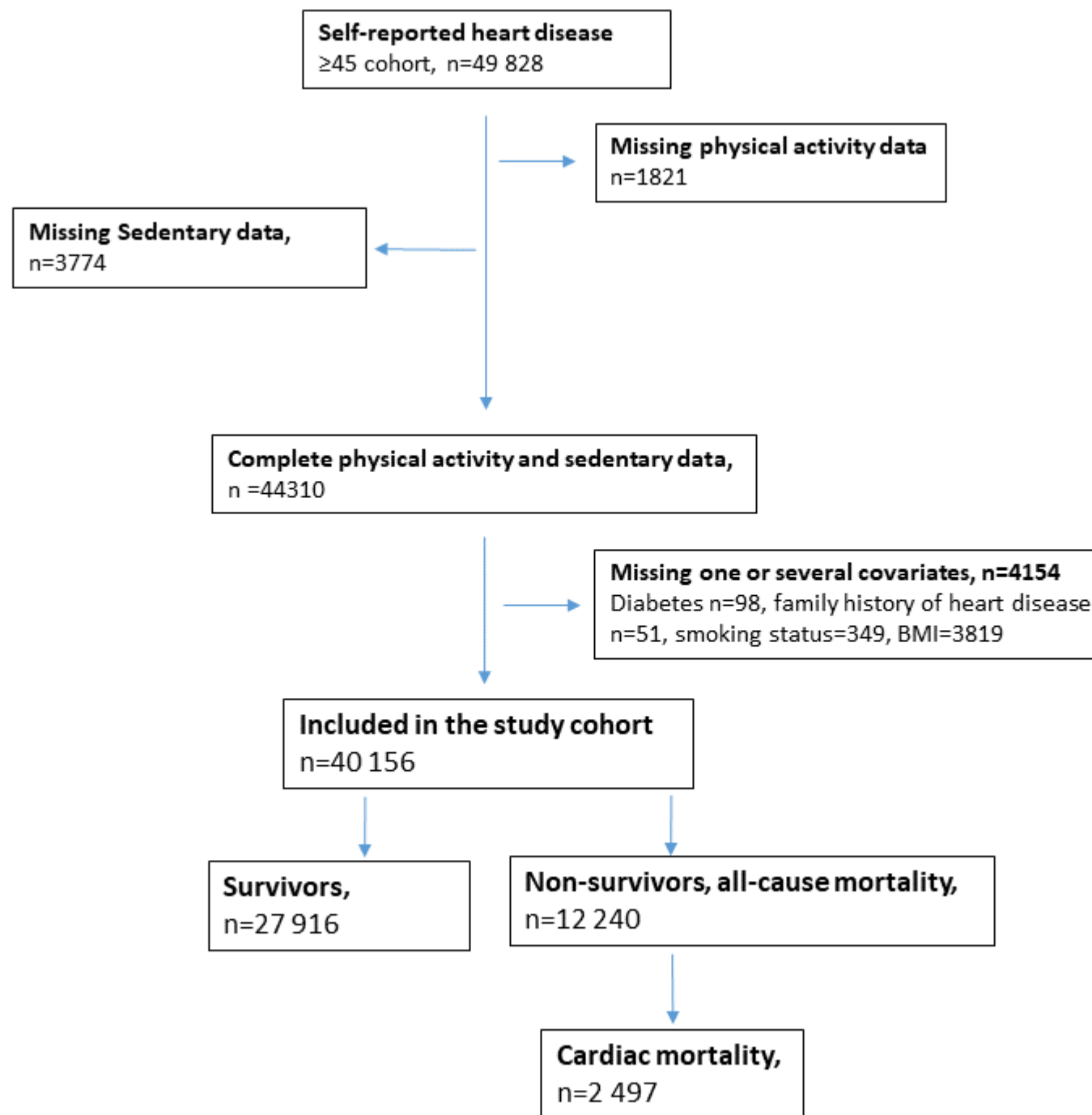

Supplement: Supplementary file 1 — Supplementary Material 1. [file 12966_2025_1743_MOESM1_ESM.pdf]
