# Supplementary material for: Developing physical Activity and Sedentary behaviour thresholds for the Secondary prevention of Heart disease (DASSH): a cohort mortality survival tree analysis
Source: Int J Behav Nutr Phys Act. 2025 Apr 10;22:43. doi: 10.1186/s12966-025-01743-6 (PMC11987228; doi:10.1186/s12966-025-01743-6)
Supplement: Supplementary file 2 — Supplementary Material 2. [file 12966_2025_1743_MOESM2_ESM.doc]

**SUPPLEMENTARY FILES**

**FIGURE LEGEND**

Supplementary Figure 1. Flow chart of included participants.

BMI, body mass index.

**TABLES**

Supplementary Table 1. Physcial Activity and Sedentary Behaviour questions asked in the 45 and Up Study.

| Questions | Wave 1 | SEEF | Wave 2 | Wave 3 |
| --- | --- | --- | --- | --- |
| Physical Activity |  |  |  |  |
| How many TIMES did you do each of these activities LAST WEEK?  *(put "0" if you did not do this activity)*  Walking continuously, for at least 10 minutes  *(for recreation or exercise or to get to or from places)*  Vigorous physical activity  *(that made you breathe harder or puff and pant, like jogging,cycling, aerobics, competitive tennis, but not household chores or gardening)*  Moderate physical activity  *(like gentle swimming, social tennis, vigorous gardening or work around the house)* | x | x | x | x |
| If you add up all the time you spent doing each activity LAST WEEK, how much time did you spend ALTOGETHER doing each type of activity (mins or hrs)?  *(put “0” if you did not do this activity)*  Walking continuously, for at least 10 minutes  *(for recreation or exercise or to get to or from places)*  Vigorous physical activity  *(that made you breathe harder or puff and pant, like jogging, cycling, aerobics, competitive tennis, but not household chores or gardening)*  Moderate physical activity  *(like gentle swimming, social tennis, vigorous gardening or work around the house)* | x | x | x | x |
| Sedentary Behaviour |  |  |  |  |
| About how many HOURS in each 24 hour DAY do you usually spend doing the following?  *(please put “0” if you do not spend any time doing it)*  Sitting  Watching television or using a computer | x | x |  |  |
| During the LAST 7 DAYS, how much time did you spend SITTING on a usual WEEK day and a usual WEEKEND day (mins or hrs):  (write your answers in the spaces provided)  for TRANSPORT (e.g. in car, bus, train etc)  at WORK (e.g. sitting at desk or using a computer)  watching TV  using a computer at home (e.g. email, games, information, chatting)  other leisure activities (e.g. socialising, movies etc but NOT including TV or computer use) |  |  | x | x |

Supplementary Table 2. Descriptive characteristics of included and excluded individuals at their baseline (first report of coronary heart disease).

| Characteristic | Excluded  (n = 9,625) | Included  (n = 40,156) |
| --- | --- | --- |
| Age *(yr)*, mean (SD) | 73.03 (10.41) | 70.25 (10.25) |
| Sex, number men (%) | 5168 (53.7) | 24878 (62) |
| Tertiary education, number (%) | 1298 (13.5) | 8682 (21.6) |
| Type 2 diabetes, number yes (%) | 2038 (21.3) | 6535 (16.3) |
| Body mass index *(kg/m^2^)*, mean (SD) | 27.14 (4.92) | 27.24 (4.71) |
| Family history heart disease, number yes (%) | 5753 (59.9) | 25,070 (62.4) |
| Current smokers, number yes (%) | 465 (5) | 1784 (4.4) |
| Sedentary behavior total (*hr/day*), median (IQR) | 5 (3-7) | 5 (3-7) |
| Moderate-to-vigorous physical activity (*min/wk*), median (IQR) | 270 (60-720) | 390 (140-840) |
| Walking (*min/wk*), median (IQR) | 60 (0-210) | 100 (30-240) |
| Moderate physical activity (*min/wk*), median (IQR) | 70 (0-300) | 120 (10-403) |
| Vigorous physical activity (*min/wk*), median (IQR) | 0 (0-00) | 0 (0-40) |
| All-cause mortality, number deaths (%) | 4,207 (43.7) | 12,240 (30.5) |
| Cardiac mortality, number deaths (%) | 898 (9.3) | 2,497 (6.2) |

Supplementary Table 3. Descriptive characteristics of survivors and non-survivors at their baseline (first report of coronary heart disease).

| Characteristic | Survivors  (n = 27,916) | Non-survivors  (n = 12,240 ) |
| --- | --- | --- |
| Age *(yr)*, mean (SD) | 67.24 (9.38) | 77.09 (8.76) |
| Sex, number men (%) | 16,474 (59) | 8404 (68.7) |
| Tertiary education, number (%) | 6916 (24.8) | 1766 (14.4) |
| Type 2 diabetes, number yes (%) | 3892 (13.9) | 2643 (21.6) |
| Body mass index *(kg/m^2^)*, mean (SD) | 27.52 (4.68) | 26.60 (4.72) |
| Family history heart disease, number yes (%) | 17,812 (63.8) | 7258 (59.3) |
| Current smokers, number yes (%) | 1204 (4.3) | 580 (4.7) |
| Sedentary behavior total (*hr/day*), median (IQR) | 4 (3-6) | 5 (4-8) |
| Moderate-to-vigorous physical activity (*min/wk*), median (IQR) | 450 (180-840) | 260 (60-660) |
| Walking (*min/wk*), median (IQR) | 120 (30-240) | 60 (0-180) |
| Moderate physical activity (*min/wk*), median (IQR) | 150 (30-420) | 90 (0-330) |
| Vigorous physical activity (*min/wk*), median (IQR) | 0 (0-60) | 0 (0-00) |

Supplementary Table 4. Hazard ratios [95% CI] for cardiac and all-cause mortality by moderate-to-vigorous physical activity and sedentary behaviour in adults with coronary heart disease (n=11,006) – sensitivity analyses.

|  | n | n cases | Unadjusted model | Adjusted model^a^ |  | Area under ROC curve^d^ | | | Sensitivity^d^ | Specificity^d^ |
| --- | --- | --- | --- | --- | --- | --- | --- | --- | --- | --- |
| Moderate-to-vigorous physical activity^b^ |  |  |  |  |  |  | | |  |  |
| All-cause mortality, <146 min/wk | 2511 | 354 | Ref | Ref |  | 0.608 | | | 0.427 | 0.212 |
| All-cause mortality, ≥146 min/wk | 8495 | 475 | 0.361 [0.315; 0.414] | 0.507 [0.440; 0.585] |  |  | | |  |  |
| Cardiac mortality, < 69 min/wk | 1649 | 38 | Ref | Ref |  | 0.607 | | | 0.362 | 0.148 |
| Cardiac mortality, ≥ 69 min/wk | 9357 | 67 | 0.277 [0.186; 0.412] | 0.465 [0.308; 0.702] |  | |  | |  |  |
| Sedentary behaviour^c^ |  |  |  |  |  |  | | |  |  |
| All-cause mortality, ≥ 0.98 hr/day | 10360 | 732 | Ref | Ref |  | 0.582 | | | 0.581 | 0.416 |
| All-cause mortality, < 0.98 hr/day | 646 | 97 | 0.426 [0.344; 0.527] | 0.850 [0.684; 1.057] |  | | |  |  |  |
| Cardiac mortality, ≥ 2.6 hr/day | 6409 | 44 | Ref | Ref |  | 0.514 | | | 0.676 | 0.648 |
| Cardiac mortality, < 2.6 hr/day | 4597 | 61 | 0.476 [0.323; 0.702] | 0.745 [0.500; 1.111] |  |  | | |  |  |

ROC, Receiver Operating Characteristic

^a^ All models adjusted for age, sex, education level, body mass index, smoking, type 2 diabetes, family history of heart disease

^b^ Model also adjusted for Sedentary Behaviour

^c^ Model also adjusted for Moderate-to-Vigorous Physical Activity

^d^ Based on the unadjusted model

Supplementary Table 5. Hazard ratios [95% CI] for cardiac and all-cause mortality using the public health recommendations for moderate-to-vigorous physical activity and sedentary behaviour in adults with coronary heart disease (n=40,156).

|  | n | n cases | Unadjusted model | Adjusted model^a^ |  | Area under ROC curve^d^ | Sensitivity^d^ | Specificity^d^ |
| --- | --- | --- | --- | --- | --- | --- | --- | --- |
| Moderate-to-vigorous physical activity^b^ |  |  |  |  |  |  |  |  |
| All-cause mortality, < 150 min/wk | 10827 | 4699 | Ref | Ref |  |  |  |  |
| All-cause mortality ≥ 150 min/wk | 29329 | 7541 | 0.514 [0.496; 0.533] | 0.659 [0.634; 0.684] |  | 0.582 | 0.384 | 0.220 |
| Cardiac mortality <150 min/wk | 10827 | 1036 | Ref | Ref |  |  |  |  |
| Cardiac mortality ≥ 150 min/wk | 29329 | 1461 | 0.455 [0.421; 0.493] | 0.623 [0.574; 0.677] |  | 0.577 | 0.415 | 0.260 |
| Sedentary behaviour^c^ |  |  |  |  |  |  |  |  |
| All-cause mortality ≥ 7 hr/day | 8592 | 3754 | Ref | Ref |  |  |  |  |
| All-cause mortality, < 7 hr/day | 31564 | 8486 | 0.808 [0.776; 0.841] | 0.820 [0.788; 0.855] |  | 0.532 | 0.259 | 0.194 |
| Cardiac mortality, ≥7 hr/day | 8592 | 699 | Ref | Ref |  |  |  |  |
| Cardiac mortality, < 7 hr/day | 31564 | 1798 | 0.724 [0.664; 0.791] | 0.750 [0.686; 0.820] |  | 0.535 | 0.280 | 0.210 |

ROC, Receiver Operating Characteristic

^a^ All models adjusted for age, sex, education level, body mass index, smoking, type 2 diabetes, family history of heart disease

^b^ Model also adjusted for Sedentary Behaviour

^c^ Model also adjusted for Moderate-to-Vigorous Physical Activity

^d^ Based on the unadjusted model
